# Designing a SARS-CoV-2 T-Cell-Inducing Vaccine for High-Risk Patient Groups

**DOI:** 10.3390/vaccines9050428

**Published:** 2021-04-24

**Authors:** Hans-Georg Rammensee, Cécile Gouttefangeas, Sonja Heidu, Reinhild Klein, Beate Preuß, Juliane Sarah Walz, Annika Nelde, Sebastian P. Haen, Michael Reth, Jianying Yang, Ghazaleh Tabatabai, Hans Bösmüller, Helen Hoffmann, Michael Schindler, Oliver Planz, Karl-Heinz Wiesmüller, Markus W. Löffler

**Affiliations:** 1 Department of Immunology, Institute for Cell Biology, University of Tübingen, Auf der Morgenstelle 15, 72076 Tübingen, Germany; cecile.gouttefangeas@uni-tuebingen.de (C.G.); sonja.heidu@uni-tuebingen.de (S.H.); juliane.walz@med.uni-tuebingen.de (J.S.W.); annika.nelde@uni-tuebingen.de (A.N.); helen.hoffmann@uni-tuebingen.de (H.H.); oliver.planz@uni-tuebingen.de (O.P.); markus.loeffler@uni-tuebingen.de (M.W.L.); 2 German Cancer Consortium (DKTK) and German Cancer Research Center (DKFZ), Partner Site Tübingen, 72076 Tübingen, Germany; ghazaleh.tabatabai@uni-tuebingen.de; 3 Cluster of Excellence iFIT (EXC2180) “Image-Guided and Functionally Instructed Tumor Therapies”, University of Tübingen, 72076 Tübingen, Germany; 4 Cluster of Excellence CMFI (EXC2124) “Controlling Microbes to Fight Infections”, University of Tübingen, 72076 Tübingen, Germany; 5 Department of Hematology, Oncology, Clinical Immunology and Rheumatology, University Hospital Tübingen, Otfried-Müller-Str. 10, 72076 Tübingen, Germany; reinhild.klein@med.uni-tuebingen.de (R.K.); beate.preuss@med.uni-tuebingen.de (B.P.); 6 German Cancer Consortium (DKTK), Clinical Collaboration Unit Translational Immunology, University Hospital Tübingen, Otfried-Müller-Str. 10, 72076 Tübingen, Germany; 7 Dr. Margarete Fischer-Bosch Institute of Clinical Pharmacology (IKP) and Robert Bosch Center for Tumor Diseases (RBCT), Auerbachstr. 112, 70376 Stuttgart, Germany; 8 Department of Oncology, Hematology and Bone Marrow Transplantation with Section of Pneumology, University Medical Center Hamburg-Eppendorf, Martinistr. 52, 20246 Hamburg, Germany; 9 Signaling Research Centres BIOSS and CIBSS, Institute of Biology III, Department of Molecular Immunology, University of Freiburg, Schänzlestr. 18, 79104 Freiburg, Germany; michael.reth@bioss.uni-freiburg.de (M.R.); yang@biologie.uni-freiburg.de (J.Y.); 10 Center for Neuro-Oncology, Comprehensive Cancer Center Tübingen-Stuttgart, University Hospital Tübingen, Hoppe-Seyler-Str. 3, 72076 Tübingen, Germany; 11 Department of Neurology & Interdisciplinary Neuro-Oncology, Hertie Institute for Clinical Brain Research, University Hospital Tübingen, Hoppe-Seyler-Str. 3, 72076 Tübingen, Germany; 12 Institute of Pathology and Neuropathology, University Hospital Tübingen, Liebermeisterstr. 8, 72076 Tübingen, Germany; hans.boesmueller@med.uni-tuebingen.de; 13 Institute for Medical Virology and Epidemiology of Viral Diseases, University Hospital Tübingen, Elfriede-Aulhorn-Str. 6, 72076 Tübingen, Germany; michael.schindler@med.uni-tuebingen.de; 14 EMC microcollections GmbH, Sindelfinger Str. 3, 72070 Tübingen, Germany; karl-heinz.wiesmueller@uni-tuebingen.de; 15 Department of General, Visceral and Transplant Surgery, University Hospital Tübingen, Hoppe-Seyler-Str. 3, 72076 Tübingen, Germany; 16 Department of Clinical Pharmacology, University Hospital Tübingen, Auf der Morgenstelle 8, 72076 Tübingen, Germany

**Keywords:** peptide vaccine, adjuvant, lipopeptide, high-risk patient, COVID-19, SARS-CoV-2

## Abstract

We describe the results of two vaccinations of a self-experimenting healthy volunteer with SARS-CoV-2-derived peptides performed in March and April 2020, respectively. The first set of peptides contained eight peptides predicted to bind to the individual’s HLA molecules. The second set consisted of ten peptides predicted to bind promiscuously to several HLA-DR allotypes. The vaccine formulation contained the new TLR 1/2 agonist XS15 and was administered as an emulsion in Montanide as a single subcutaneous injection. Peripheral blood mononuclear cells isolated from blood drawn before and after vaccinations were assessed using Interferon-γ ELISpot assays and intracellular cytokine staining. We detected vaccine-induced CD4 T cell responses against six out of 11 peptides predicted to bind to HLA-DR after 19 days, following vaccination, for one peptide already at day 12. We used these results to support the design of a T-cell-inducing vaccine for application in high-risk patients, with weakened lymphocyte performance. Meanwhile, an according vaccine, incorporating T cell epitopes predominant in convalescents, is undergoing clinical trial testing.

## 1. Introduction

After the severe acute respiratory syndrome coronavirus 2 (SARS-CoV-2), causing the coronavirus disease 2019 (COVID-19), was first sampled and described in December 2019 in Wuhan, China [1] and declared a pandemic by the World Health Organization on 11 March 2020 [2], the global scientific community responded to this urgency in several resourceful ways. For example, hitherto unapproved approaches and vaccine platforms initially intended for immune oncology applications and other diseases were swiftly repurposed, which enabled the development of COVID-19 vaccines at an unprecedented pace [3,4]. Meanwhile various vaccines to prevent COVID-19 are approved and are available all over the world. However, appropriate vaccines for specifically inducing T cell responses are still lacking. Currently, for most approved vaccines, evaluations of immune correlates for protection primarily focused on humoral immune responses, providing only very limited data on T cell induction [5].

Thus far, millions of people worldwide are affected by SARS-CoV-2 in part with devastating consequences and high mortality rates are found among high-risk populations, including the elderly [6] and patients with co-morbidities [7], such as cancer [8]. Particularly for specific patient groups, the hitherto available vaccines might demonstrate only limited efficacy or require additional T cell induction, e.g., in patients with B cell malignancies treated with depleting antibodies.

Another legitimate way to gain preliminary evidence for novel treatments is self-experimentation, a practice with historical importance that is only permissible under very specific conditions and with strict restrictions that need to be considered in any event. It should be respected that the practice remains restricted to single volunteers that can fully oversee the implications of their actions and must be performed in absence of any coercion or dependency [9,10]. In such a context and considering the current COVID-19 urgency, this practice might yield valuable preliminary research results and increase public trust [11] but must be performed in a responsible way and cannot avoid formal clinical trial testing.

**Figure 1:**
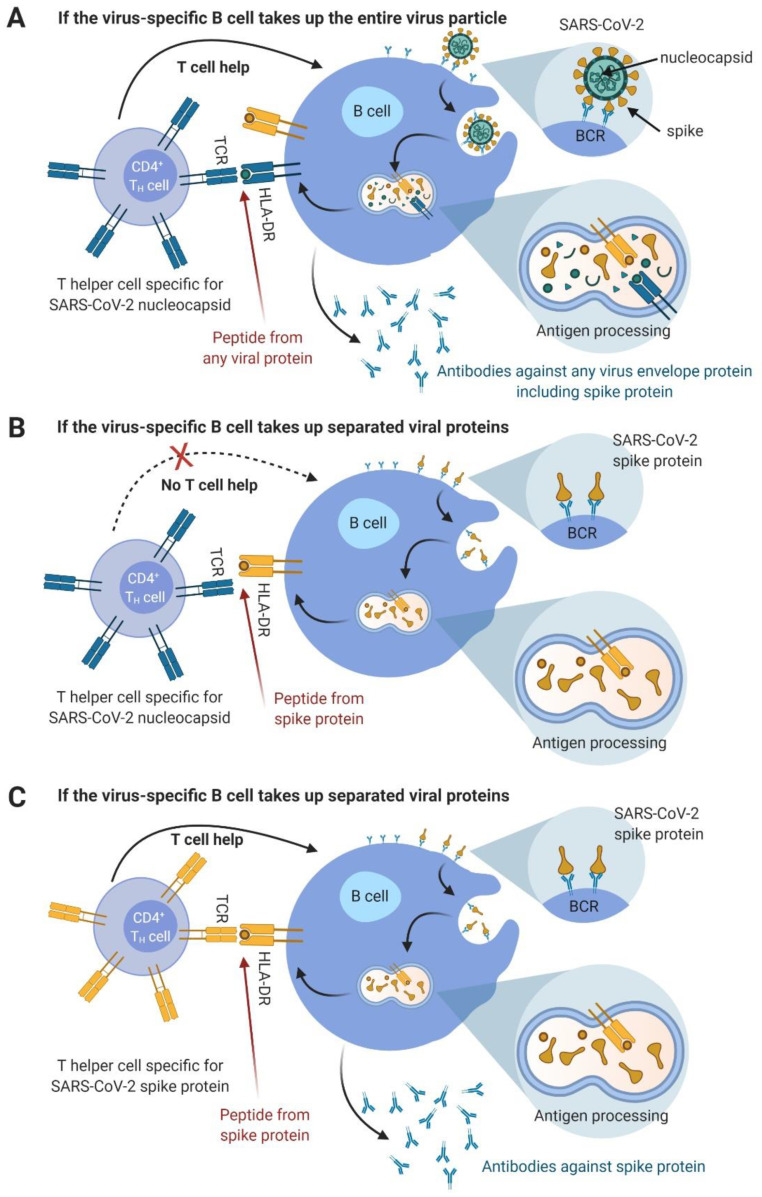
Illustration of the potential interactions between SARS-CoV-2 and B and T cells. (**A**) If a virus-specific B cell takes up the entire virus particle via B cell receptor (BCR)-mediated phagocytosis, all structural viral proteins should be processed in the HLA class II processing vesicle. The resulting peptides should be loaded onto the HLA class II molecules. For a review, see Avalos et al. (2014) [14]. Only a selection of the resulting peptides fit to the respective HLA molecules present, based on their peptide specificity. The B cell will then present these peptides on the cell surface, with one peptide per HLA molecule. If a T cell is specific for exactly this peptide–HLA combination and if the B cell is activated via its BCR–antigen contact, then the CD4 T helper cell would deliver help to this B cell, both through cellular interaction and cytokines. Since all viral proteins are presented on the B cell’s HLA in this scenario, a nucleocapsid-specific T cell also activates a spike-specific B cell. (**B**) If the virus-specific B cell takes up separate viral proteins via BCR-mediated phagocytosis, e.g., after previous destruction of viral particles by follicular dendritic cells, only peptides from these proteins are presented on the B cell’s HLA molecules. This is shown here for the spike glycoprotein. Thus, a nucleocapsid-specific T cell does not activate a spike-specific B cell in this constellation. (**C**) If the same B cells as described in (**B**) are activated by a spike-specific CD4 T helper cell, the B cell is now activated. Abbreviations: BCR—B cell receptor, HLA—human leucocyte antigen, SARS-CoV-2—severe acute respiratory syndrome coronavirus 2, TCR—T cell receptor. Figure was designed with BioRender.

We previously reported the results of an experimental approach based on a peptide vaccine consisting of various viral epitopes. Peptides were administered together with Montanide and a novel lipopeptide adjuvant, the toll-like receptor (TLR) 1/2 ligand XS15, to the same healthy volunteer [10]. Building on this previous work, we here report the findings from self-experimentation, involving the same volunteer from March and April 2020 [12]. The previously tested protocol for vaccination was used, but this time the vaccine contained in silico predicted peptide sequences from SARS-CoV-2. Relevant data that were available very early from a virus isolate from China, were used for this purpose [13] and the peptide selection was completed on 23 January 2020. The design of the current vaccine included the careful selection of candidate epitopes that are promiscuous for most human leukocyte antigen (HLA)-DR allotypes (theoretical considerations are illustrated in Figure 1). Meanwhile early phase clinical trial testing for a respective vaccine is ongoing (clinicaltrials.gov—registration number: NCT04546841), which also incorporates the knowledge that T cell responses alone are found in a fraction of convalescents.

## 2. Materials and Methods

### 2.1. SARS-CoV-2 Peptide Prediction and Selection

The sequences described here were derived from a virus isolate obtained from a worker at the Wuhan fish market (https://www.ncbi.nlm.nih.gov/nuccore/MN908947, accessed on: 22 January 2020, [13]). Using the SARS-CoV-2 nucleocapsid and envelope protein sequences from this isolate and the prediction software SYFPEITHI (www.syfpeithi.de, accessed on: 22 January 2020, [15]), we predicted candidate CD8 (HLA-A*01 or -B*08) and candidate CD4 T cell epitopes (HLA-DRB1*11) based on the HLA allotype of the healthy volunteer. We chose the nucleocapsid protein, because nucleoprotein peptides from many other viruses are potent T cell epitopes (see: www.iedb.org, accessed on: 22 January 2020). Peptide selection for the first vaccination was completed on 23 January 2020. We synthesized eight peptide sequences that had the highest SYFPEITHI-scores and no cysteine residues (for chemical stability reasons). They were complemented by two CMV-derived peptides that had previously elicited immune responses in the same individual and could thus be used as positive controls [10]. These ten peptides (Table 1) were synthesized in-house using automated peptide synthesis.

For the prediction of promiscuous peptides [16] for the second vaccination, the ORF protein sequences were split into peptides of 15 amino acids. The prediction algorithm SYFPEITHI 1.0 was used to predict the binding to HLA-DRB1*01:01, -DRB1*03:01, -DRB1*04:01, -DRB1*07:01, -DRB1*11:01, and -DRB1*15:01. The 5% top-scoring peptides of each protein (based on the total length of each ORF) and each HLA-DR allotype were selected. Position-based sorting of peptides within each protein revealed peptide clusters of promiscuous peptides binding to several HLA-DR allotypes. Through cluster-based selection, peptide clusters of promiscuous peptides with a common core sequence of 9 amino acids were selected. Thereby, 8 and 2 clusters were chosen for the nucleocapsid and the spike protein. Of each selected cluster, one representative peptide was selected for vaccination, excluding any cysteine containing peptides (Table 2).

### 2.2. Self-Administered Vaccination

The first (personalized) vaccine consisted of ten peptides (see Table 1) solubilized in water and 20% dimethyl sulfoxide (DMSO). It further contained the lipopeptide adjuvant XS15 (50 μg) and was administered as an emulsion with an equal volume of Montanide™ ISA51 VG (Seppic, Paris, France). A total volume of 0.5 mL was administered subcutaneously (s.c.) under the skin of the left side of the abdomen, on 6 March 2020. The vaccinated individual did not report any flu-like symptoms or other symptoms consistent with SARS-CoV-2 infection during the relevant period. The individual further did not report visiting any areas designated as high risk for virus transmission. The second vaccination was applied in the same way on the right side of the abdomen on 3 April 2020.

### 2.3. Ethical and Scientific Considerations

As previously reported in a comparable self-vaccination study by the same volunteer [10], we considered this to be an ethically and legally legitimate form of experimentation [9]. Self-experimentation is a special case of research and limited to individual subjects. It is essential that such self-experimentation does not contravene any personal interest or ethical imperative. Coercion and dependency could be excluded here, ensuring decision-making autonomy. The volunteer in this case was a renowned expert in immunology and thus was able to understand any risks and implications of his own actions. It should therefore be considered that this study was permissible. In addition, relevant precedent exists, where human self-experimentation opened up new avenues for research and contributed to medical progress [17]. We are naturally aware that single case reports cannot provide conclusive evidence or any generalizable results. Rather these findings enable the formulation of new hypotheses. Case reports are attributed with a high sensitivity for detecting novelty and are deemed relevant for medical progress [18]. This study was intended as a starting point for intensified discussion and development, rather than a substitute for proper drug development and clinical trials. Meanwhile, a phase I clinical trial using promiscuous SARS-CoV-2 HLA-DR binding peptides, together with the adjuvant XS15 was initiated after receiving the required regulatory and institutional review board approvals.

### 2.4. Immunomonitoring

Peripheral blood mononuclear cells (PBMCs) were isolated from heparin-anticoagulated peripheral venous blood drawn before and after vaccinations, via density centrifugation [19], and were either frozen and stored in liquid nitrogen or used fresh. In addition, plasma diluted 1:1 with phosphate buffered saline (PBS) was obtained during PBMC isolation. Blood serum from three healthy blood donors (HDs) was obtained after informed consent for use as control samples. For in vitro amplification (IVS) of vaccine-specific cells, PBMCs were seeded in a 24-well plate in T cell medium (TCM; i.e., IMDM with 10% heat-inactivated human serum, 100 units/mL penicillin, 0.1 mg/mL streptomycin, and 50 μM β-mercaptoethanol) and cultured overnight at 37 °C, 7.5% CO_2_. On day 1, peptides were added in pools at 10 or 5 μg/mL each for HLA class I and class II peptides, respectively, and interleukin-2 (rIL-2, R&D Systems, Minneapolis, MN) was added to the culture on days 3, 5, 7, and 9. Cells were harvested on day 12, counted, and analyzed either with intracellular cytokine staining (ICS) or Enzyme Linked ImmunoSpot (ELISpot) assay [19]. For ex vivo interferon-γ (IFNγ) ELISpot, PBMCs obtained pre-vaccination were thawed, while those obtained post-vaccination were used directly ex vivo after density gradient separation. All cells were rested overnight in a culture medium (IMDM with 10% heat-inactivated human serum, 100 units/mL penicillin, 0.1 mg/mL streptomycin, and 50 μM β-mercaptoethanol), containing 1 μg/mL DNase I before starting the ELISpot. A total of 300,000 cells per well were plated on a pre-coated ELISpot plate. The negative control (DMSO in water) and vaccine peptides were tested in six and three replicates, respectively. For phytohemagglutinin (PHA-L) stimulation (positive control), duplicates were plated with 150,000 cells per well. For IFNγ ELISpot analyses following in vitro 12-day expansion, 100,000 cells were used per well. Peptides were added at a concentration of 5 μg/mL (for HLA class I peptides) or 2.5 μg/mL (for HLA-DR peptides), and PHA-L was used at 10 μg/mL. The IFNγ ELISpot was performed as described previously [10,19], with incubation at 37 °C in a 7.5% CO_2_ atmosphere for 26 h before development. Spot measurement was performed on an ImmunoSpot series 6 ultra-V analyzer (CTL Europe GmbH, Bonn, Germany). For the intracellular cytokine assay, cells obtained after the 12-day culture were plated at 1 × 10^6^ cells/well in a 96-well plate and cultured for 14 h in the presence of Brefeldin A (10 μg/mL, Sigma-Aldrich, St. Louis, MO) and Golgi Stop (BD Biosciences, Heidelberg, Germany), and 10 μg/mL of the individual peptides or the equivalent amount of DMSO/water. Staphylococcus enterotoxin B (Sigma-Aldrich) was used at 10 μg/mL as the positive control. Cells were thereafter stained with Zombie Aqua live dead, CD4 APC-Cy7 (both Biolegend, San Diego, CA) and CD8 PE-Cy7 (Beckman Coulter, Krefeld, Germany) antibodies, permeabilized with Cytoperm/Cytofix (BD Biosciences), then stained intracellularly with the following specific antibodies—IFNγ FITC, IL-2 PE (both BD Biosciences), CD154 APC, and TNF Pacific Blue (both Biolegend). All cells were acquired on a flow cytometer (LSR Fortessa, BD Biosciences) and analyses were performed with FlowJo (FlowJo, Ashland, OR) [10,19]. Antibody reactivity in the plasma obtained from the vaccinated volunteer and in serum from three HDs (for comparison) was used in an in-house ELISA performed as described previously [19]. In brief, 96-well plates were incubated for 16 h with the respective peptides (produced in-house at the University of Tübingen, Department of Immunology, Tübingen, Germany) or with recombinant proteins (SinoBiological, Beijing, China), utilized at a concentration of either 35 μg/mL or 1 μg/mL. Plasma or serum samples were diluted 1:500 (previously determined to be optimal for the analysis of peptide–antibody reactivity). To differentiate between IgG- and IgM-antibodies, peroxidase-conjugated goat anti-human IgG and IgM-antibodies were applied in parallel. Ortho-phenylene-diamine was used as a substrate. The reactivity was measured with an ELISA-reader at 450 nm and was expressed as the optical density multiplied by 1000 (OD × 10^3^). Cut-off values were determined by testing sera from healthy blood donors against the respective antigens/peptides. Values above the mean of OD × 10^3^ plus twice the standard deviation were defined as antibody positive.

## 3. Results

### 3.1. Clinical Aspect of the Vaccination Sites

The first vaccine with the peptides shown in Table 1 was administered subcutaneously (s.c.) under the skin of the left abdomen on 6 March 2020, the second with the peptides shown in Table 2 on the right-hand side on 3 April 2020. As expected with any s.c. vaccination using Montanide [10,20] or when further adding XS15 as an adjuvant [10], a granuloma developed in both cases. These were palpable from day one and grew to a maximum size of approximately 3 × 5 × 1.5 cm by day 12. In this case, the skin surface temperature was 37.0 °C at the center of the granuloma vs. 36.2 °C on adjacent visually unaffected abdominal skin. The granulomas were described as painless, slightly itchy indurations that were sensitive to touch, particularly between days 9 and 14, and started to shrink in size from day 13. No other adverse effects were noticed by the vaccinated volunteer, particularly no systemic adverse reactions to the vaccine.

### 3.2. T Cell Responses

**Figure 2:**
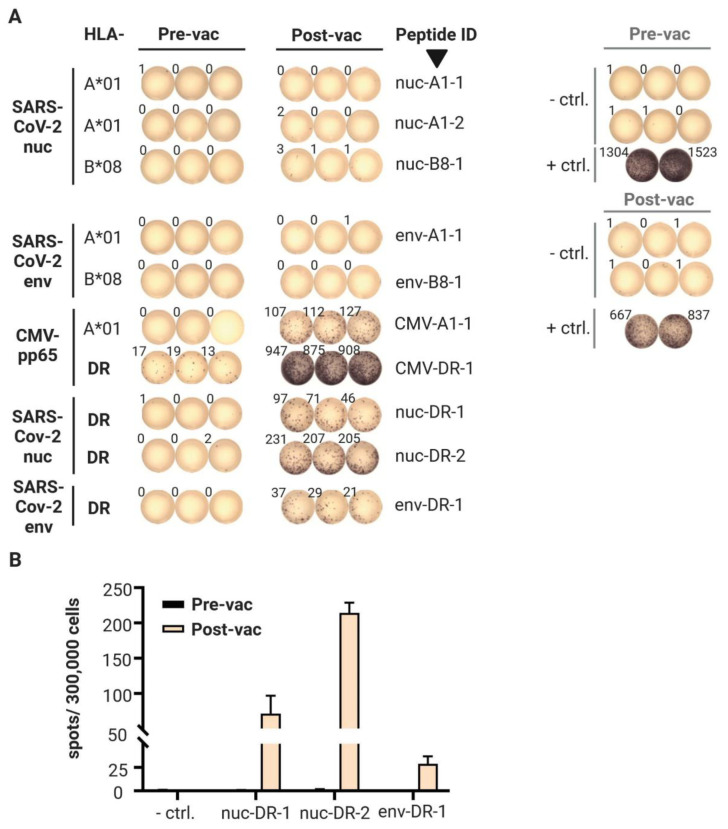
Results of the ex vivo Interferon-γ ELISpot after the first vaccination. (**A**) ELISpot wells showing spot counts obtained from pre- and post-vaccination PBMC samples tested against each of the specified vaccine peptides (*n* = 10, in triplicate). The negative control (ctrl.—DMSO and water) was tested in six replicate wells and the positive control (+ ctrl.—phytohemagglutinin) in duplicate wells. The images representing the ELISpot wells were rearranged for this illustration. (**B**) Graph of spot numbers for the three SARS-CoV-2-derived HLA-DR peptides. The mean and SD are shown from three technical replicates. Abbreviations: CMV—cytomegalovirus, ctrl.—control, DMSO—dimethyl sulfoxide, env—envelope protein, nuc—nucleoprotein, pre-vac—36 days before vaccination, post-vac—19 days after vaccination, and SARS-CoV-2—severe acute respiratory syndrome coronavirus 2.

First vaccination: Ex vivo IFNγ ELISpot results from pre- and post-vaccination PBMCs are shown in Figure 2A,B. The CMV-pp65 HLA-DR epitope YQEFFWDANDIYRIF (amino acids 510–524; CMV-DR-1), which was included as a positive control, was weakly recognized (mean: 16 spots/300,000 cells; background < 1) before this vaccination. Notably, three years prior, vaccination with this peptide resulted in a mean spot count of 525 four weeks after the peptide was administered, with the same adjuvant and protocol [10]. As expected from our previous experience, boosting with one additional vaccination more than one year later with the same peptide gave a strong response (mean: 910 spots/300,000 cells). The CMV-pp65 HLA-A*01 epitope YSEHPTFTSQY (amino acids 363–373; CMV-A1-1), which induced a weak response in an ex vivo ELISpot three years prior with a mean spot count of only 12, and showed a negative response before the recent booster vaccination, gave a mean spot count of 115. These results indicated that a three-year memory against these HLA class II- and class I-restricted CMV-pp65 epitopes persisted after a single vaccination. Importantly, the vaccinated volunteer was previously tested as CMV seronegative. No pre-existing SARS-CoV-2-directed T cell responses were detectable prior to this first SARS-CoV-2 vaccination. The response to the five predicted SARS-CoV-2 HLA class I peptides remained negative after vaccination. In the meantime, we know that our predictions did not find the immunodominant HLA class I epitopes recognized by the T cells of convalescents [16], and thus, might not be immunogenic at all. In contrast, a strong T cell response was induced against all three SARS-CoV-2 HLA class II peptides by a single vaccination (Figure 2). Thus, all three SARS-CoV-2-derived peptides predicted for HLA-DR-binding induced T cells to produce IFNγ, most probably representing a Th1 response. Phenotyping of the responding T cell subsets and the production of further cytokines by intracellular cytokine staining confirmed this notion (Table 3).

**Figure 3:**
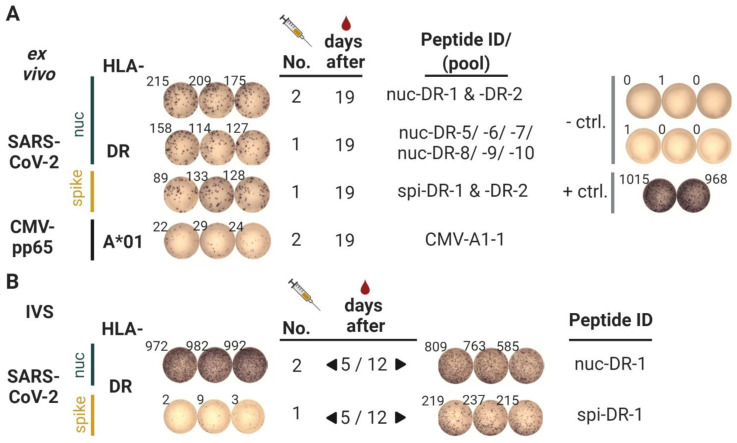
Results of the Interferon-γ ELISpot after the second vaccination. (**A**) PBMCs were assayed by ex vivo interferon-γ ELISpot. Wells showing spot counts from post-vaccination PBMC samples obtained from blood drawn 19 days after the second vaccination. The specified peptide pools, consisting of several of the vaccinated peptides, were either vaccinated before (boost; second vac) or vaccinated for the first time (after first vaccination); tests were performed in triplicates. The negative control (- ctrl.—DMSO and water) was tested in six replicate wells and the positive control (+ ctrl.—phytohemagglutinin) in duplicate wells. The images representing the ELISpot wells were rearranged for this illustration. (**B**) PBMCs were assayed by interferon-γ ELISpot (100.000 cells/well), after 12 days of in vitro expansion in the presence of the relevant peptides (in vitro stimulation; IVS). PBMCs were isolated from blood drawn 5 days (left side) or 12 days (right side) after the second vaccination; peptides were either vaccinated before (boost; second vaccination) or newly vaccinated for the first time (after first vac), as indicated by the column number. Figure was designed with BioRender.

Second vaccination: Strong ex vivo T cell responses could be detected at day 19, when testing peptides in pools (Figure 3A). The three pools with nucleoprotein and spike peptides showed a strong response. Two 9-mer-peptides embedded in 15-mer peptides (VYAWNRKRI, SVLYNSASF) were recognized by CD4 T cells after one round of in vitro peptide stimulation (Table 3). The two new spike peptides were also tested with blood drawn at days 5 and 12, respectively, after in vitro stimulation, to get an idea about the kinetics of T cell induction. As indicated in Figure 3B, no response was detectable on day 5 against the spike peptide ASVYAWNRKRISN, but could be evidenced later on day 12. In order to resolve the peptide specificity of all induced T cell responses, as well as the functional phenotype of the T cells, intracellular stainings were performed for all peptides used (Table 3). Thus, all CD4 T cells induced produce IFNγ, IL-2, CD154, and TNF. The two boost peptides from nucleocapsid and the newly vaccinated spike peptide ASVYAWNRKRISN (spi-DR-1) showed especially strong responses, as well as the nucleocapsid peptide LLLLDRLNQLESKMS (nuc-DR-7). Since the latter was included in a peptide-pool tested with PBMCs from a pre-vaccination time-point (23 March 2021), which showed some T cell reactivity, this strong reaction could also be a boost reaction of a naturally existing cross-reactive T cell response. Indeed, we found T cells against this peptide in other non-infected individuals [16]. One of the spike peptides (VADYSVLYNSASFST; spi-DR-2) was not recognized as a 15-mer, but as an embedded 9-mer peptide (SVLYNSASF), it did show responses (Table 3 and ELISpot; ELISpot results not shown). Taken together, after vaccination with ten promiscuous HLA-DR peptides, we could detect T cell responses against five peptides, plus an embedded peptide.

### 3.3. Antibody Responses

Antibodies contained in the plasma of the volunteer and serum of three healthy blood donors were tested using an ELISA. IgG and IgM antibodies against the three SARS-CoV-2 CD4 peptides were not detectable or showed negligible induction (Table S1). We know from previous work that repeated vaccination with CD4 peptides in Montanide with or without additional adjuvants leads to the induction of antibodies [19]. We, therefore, speculate that antibodies against these epitopes might develop later on. There seems to be a weak recall IgG response against the CMV-pp65 CD4 epitope YQEFFWDANDIYRIF (an 8-fold increase compared to the reactivity before vaccination). It is interesting that two out of the three healthy donors showed high IgG reactivity against YQEFFWDANDIYRIF (amino acids 510–524; CMV-DR-1). Testing serum for antibodies against linear synthetic peptides from all proteins, not only from the surmised neutralizing epitope-bearing ones, might be a useful complement to the use of recombinant proteins. In addition, sera of the vaccinated volunteer and several healthy donors, obtained before the current pandemic, were tested with an ELISA covering SARS-CoV-2 proteins and peptides (Table S2). Further, serum from one donor (HD CoV+) who was tested positive for SARS-CoV-2 according to medical routines (qRT-PCR) and recovered from the infection, was included. This serum showed strong reactivity against the spike protein, but not the serum from the vaccinated volunteer, indicating that the latter was not infected.

## 4. Discussion

After the first vaccination, ex vivo IFNγ-ELISpot results revealed strong T cell responses against all three SARS-CoV-2-derived CD4 peptides, 19 days after a single s.c. vaccination with the peptides, Montanide and the TLR 1/2 ligand XS15. Based on previous findings, we assumed that these immune responses were most probably robust and durable [10]. There were no detectable T cell responses against the five CD8 peptides and no measurable antibody responses. In the second vaccination, ten CD4 peptides were used, two from spike and eight from the nucleocapsid. T cell responses against six of these were induced, starting to be detectable 12 days after vaccination. Based on these findings, we developed a SARS-CoV-2 peptide vaccine that was particularly intended for persons who tend to have weaker CD4 T cell activity, e.g., patients with B cell malignancies, meanwhile undergoing early phase clinical trial testing. The potential of this approach is based on the following assumptions.

1. CD4 Th1 cells should vigorously activate virus antigen-experienced B cells that should already pre-exist in most COVID-19-patients. An illustration for this assumption is provided in Figure 1. These CD4 T cells would be expected to directly contribute to virus clearance and deliver strong T helper signals to the CD8 T cells already primed during natural infection. The resulting enhanced activity could lead to more rapid virus clearance or transiently increased lung damage.
2. Vaccine-induced CD8 T cells against peptides embedded in the longer peptides do appear later, as preliminary data from the majority of subjects in our presently running study suggest (https://clinicaltrials.gov, accessed on 20 April 2021, Identifier: NCT04546841). In this study, six immunodominant 15-mer peptides reported in [16] are used in a vaccine with Montanide and XS15. Once activated, such CD8 T cells should also contribute to faster virus clearance.
3. Vaccine-induced antibodies against the viral peptides tested in this study might also appear much later, if at all.
4. Since we found IFNγ-producing T cells, we conclude that Th1 CD4 T cells were induced. Therefore, there should be no disease enhancing-effects related to the induction of Th2-bias, as described for other coronaviruses [21].

**Figure 4:**
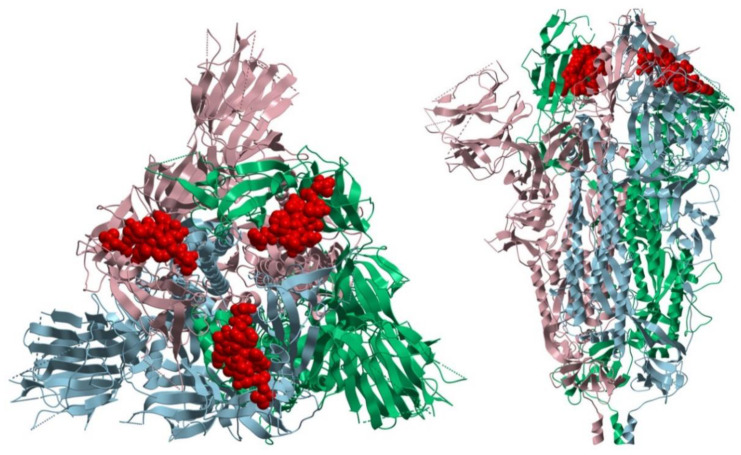
In silico prediction of the localization of a CD4 candidate epitope within the SARS-CoV-2 spike protein. Ribbon representation of the top view (**left**) and side view (**right**) of the SARS-CoV-2 spike ectodomain in its closed state (PDB:6VXX) with the peptide VADYSVLYNSASFST highlighted in the Red Corey-Pauling-Koltun (CPK) model. Image was produced using the Molsoft ICM-Browser.

Of course, a vaccine designed for broad use must be designed to be suitable for all patients independent of their individual HLA allotypes. Based on the experiments described here, and our recent analysis of T cell responses in 180 SARS-CoV-2 infected individuals [16], we proposed that SARS-CoV-2 peptides that are recognized frequently by CD4 T cells in this cohort should be used for a first exploration of this vaccine concept. From spike glycoprotein, only those peptides should be included that are not exposed on the virus particle and thus should not be recognizable by antibodies. This could be judged by in silico prediction of the site of candidate peptides within the spike protein (see Figure 4). We think this measure should exclude any risk of inducing antibody-dependent enhancement (ADE) through non-neutralizing antibodies improving viral uptake, as was described for SARS-CoV-1 and dengue virus infection in humans and feline infectious peritonitis virus in cats [22,23,24,25]. Thus, our strategy was to carefully select spike glycoprotein-derived candidate epitopes that were promiscuous for most HLA-DR allotypes, recognized by infected people, but that did not represent any potential B cell epitopes, as far as could be judged using in silico analysis and analysis of serum antibodies from SARS-CoV-2-infected patients. The relevant underlying theoretical considerations are illustrated in Figure 1.

Vaccination with T cell epitopes alone should be suitable for prophylactic as well as therapeutic vaccination. As the situation in zoonotic coronaviruses suggests, this strategy might even be superior to traditional vaccination approaches, where primarily B cell responses against the vaccine antigens are to be induced, since in many such infections mainly memory T cells and not antibodies seem to play the major role for long-term-immunity against disease [26]. A vaccine solely consisting of T cell epitopes would not necessarily be expected to completely prevent infection, as with traditional vaccines aiming for strong neutralizing antibody responses. However, it would help the patient’s immune system to quickly resolve the commencing infection by fostering faster antibody production, enabled by the vaccine-induced CD4 T cells. On the other hand, a fraction of SARS-CoV-2 convalescents do not show any antibody responses, indicating clearly that T cells alone could be protective [16].

Induction of T cells against several viral proteins should also have the advantage of protection against virus mutations affecting only one viral protein, as was discussed in the context of influenza [27]. In the case of SARS-CoV-2, this type of vaccine should also work for new viral serotypes and variants that already exist in so far unknown environments or could develop in the future. Taken together, we propose an immunization strategy that induces T cells but indirectly affects B cells, to be applied in at-risk patients before or after SARS-CoV-2 infection. This might be especially beneficial for older individuals who frequently have lower numbers of CD4 T cells, and for cancer patients with treatment-induced lymphopenia. A phase I clinical study based on these considerations was started in November 2020 (EudraCT Number: 2020-002502-75) in healthy young individuals.

## Figures and Tables

**Table 1:** Peptides included in the first vaccination (6 March 2020).

AA Sequence	Predicted HLA Restriction	Source Protein	Peptide ID	AA Position	Peptide Length	Administered Amount (μg)
SPDDQIGYY	A*01	SARS-CoV-2 nuc	nuc-A1-1	79–87	9	240
MKDLSPRWY	A*01		nuc-A1-2	101–109	9	240
LLLDRLNQL	B*08		nuc-B8-1	222–230	9	240
IGYYRRATRRIRGGD	DR		nuc-DR-1	84–98	15	240
ASAFFGMSRIGMEVT	DR		nuc-DR-2	311–325	15	240
VSLVKPSFY	A*01	SARS-CoV-2 env	env-A1-1	49–57	9	720
YVYSRVKNL	B*08		env-B8-1	57–65	9	720
FYVYSRVKNLNSSRV	DR		env-DR-1	56–70	15	240
YSEHPTFTSQY	A*01	CMV-pp65	CMV-A1-1	363–373	11	720
YQEFFWDANDIYRIF	DR		CMV-DR-1	510–524	15	240

Vaccination was performed with synthetic peptides solubilized in water and 20% DMSO, including 50 μg XS15 as an adjuvant, emulsified in Montanide ISA51 VG in a total volume of 0.5 mL. The varying dosing of peptides (240 μg for all HLA class II_restricted peptides and 720 μg for some of the HLA class I-restricted peptides) was used to address technical questions, based on speculations that a higher antigen load might induce better CD8 T cell responses for HLA class I-restricted peptides in this particular vaccine approach. Abbreviations: AA—amino acid, CMV—cytomegalovirus, DMSO—dimethyl sulfoxide, env—envelope protein, HLA—human leukocyte antigen, and nuc—nucleoprotein.

**Table 2:** Peptides from SARS-CoV-2 predicted to bind to multiple HLA-DR molecules included in the second vaccination (3 April 2020).

AA Sequence	Source Protein	Peptide ID	AA Position	Peptide Length
ASVYAWNRKRISN	SARS-CoV-2 spike	spi-DR-1	348–360	13
VADYSVLYNSASFST		spi-DR-2	362–376	15
*IGYYRRATRRIRGGD*	SARS-CoV-2 nuc	nuc-DR-1	84–98	15
*ASAFFGMSRIGMEVT*		nuc-DR-2	311–325	15
RWYFYYLGTGPEAGL		nuc-DR-5	107–121	15
ASWFTALTQHGKEDL		nuc-DR-6	50–64	15
LLLLDRLNQLESKMS		nuc-DR-7	221–235	15
AADLDDFSKQLQQSM		nuc-DR-8	397–411	15
AIVLQLPQGTTLPKG		nuc-DR-9	156–170	15
YKHWPQIAQFAPSAS		nuc-DR-10	298–312	15

Vaccination was performed with synthetic peptides solubilized in water and 20% DMSO including 50 μg XS15 as an adjuvant, emulsified in Montanide ISA51 VG in a total volume of 0.5 mL. Peptides already used in the first vaccination (6 March 2020) (boost) are shown in italics. The peptide amount administered in the vaccine was 240 μg for each peptide. Abbreviations: AA—amino acid, DMSO—dimethyl sulfoxide, HLA—human leukocyte antigen, and nuc—nucleoprotein.

**Table 3:** Intracellular cytokine staining of CD4+ PBMCs drawn 19 days after the second vaccination, following in vitro stimulation with the respective peptides.

AA Sequence	Source Protein	Peptide ID	IFNγ	IL-2	CD154	TNF
			% (Fold Increase)			
IGYYRRATRRIRGGD	nuc	nuc-DR-1	**4.2 (199)**	**1.6 (35)**	**16.0 (60)**	**14.2 (191)**
ASAFFGMSRIGMEVT	nuc	nuc-DR-2	**12.0 (571)**	**3.2 (67)**	**31.7 (118)**	**24.0 (321)**
AS*VYAWNRKRI*SN	spike	spi-DR-1	**8.9 (424)**	**4.6 (76)**	**34.0 (122)**	**22.0 (201)**
VADY*SVLYNSASF*ST	spike	spi-DR-2	0.0	0.0	−0.3	−0.1
RWYFYYLGTGPEAGL	nuc	nuc-DR-5	**0.1 (4)**	**0.3 (6)**	**1.3 (4)**	**1.2 (12)**
ASWFTALTQHGKEDL	nuc	nuc-DR-6	0.0	0.0	0.1	0.1
LL*LLDRLNQL*ESKMS	nuc	nuc-DR-7	**16.0 (696)**	**3.3 (62)**	**37.1 (99)**	**31.1 (312)**
AADLDDFSKQLQQSM	nuc	nuc-DR-8	0.0	0.0	−0.1	0.0
AIVLQLPQGTTLPKG	nuc	nuc-DR-9	0.0	0.0	0.0	−0.1
YKHWPQIAQFAPSAS	nuc	nuc-DR-10	0.0	0.0	−0.1	0.0
Embedded AA sequence						
VYAWNRKRI *	spike	-	**1.6 (45)**	**1.0 (13)**	**15.8 (13)**	**8.0 (22)**
SVLYNSASF *	spike	-	**4.6 (128)**	**3.9 (49)**	**17.6 (15)**	**15.1 (41)**
LLDRLNQL *	nuc	nuc-B8-1	0.0	**0.2 (3)**	0.2	0.4

Embedded sequences * tested are shown in italics. Percentages of marker+ cells are given within the living CD4+ lymphocytes, after subtraction of the values found in the unstimulated cells (neg. ctrl.—DMSO/water). T cell responses (given in bold) were defined for each marker as: (i.) frequency of marker-positive CD4+ [peptide stimulation] at least 3-fold higher than the frequency of marker-positive CD4+ cells [neg. ctrl.], and (ii.) at least 20 marker-positive CD4+ events in the peptide stimulated condition. Mean % positive cells in the unstimulated control samples were 0.02%, 0.06%, 0.55%, and 0.17% for IFNγ, IL-2, CD154, and TNF, respectively; fold increase over control is indicated in brackets. Abbreviations: AA—amino acid, neg. ctrl.—negative control, IFNγ—interferon-γ, IL—interleukin, nuc—nucleoprotein, and TNF—tumor necrosis factor.

## Data Availability

Data relating to this study are either provided in this article or available as supplementary materials. Any additional relevant information or source data may be requested from the corresponding authors.

## Supplementary Materials

The following are available online at https://www.mdpi.com/article/10.3390/vaccines9050428/s1. Table S1: Antibody responses in a healthy self-vaccinated volunteer and healthy blood donors without anamnestic SARS-CoV-2 exposure. Table S2: Antibody responses in a healthy self-vaccinated volunteer and healthy blood donors with confirmed and without SARS-CoV-2 exposure.
