# Supplementary material for: Designing a SARS-CoV-2 T-Cell-Inducing Vaccine for High-Risk Patient Groups
Source: Vaccines (Basel). 2021 Apr 24;9(5):428. doi: 10.3390/vaccines9050428 (PMC8146137; doi:10.3390/vaccines9050428)
Supplement: Supplementary file 1 [file vaccines-09-00428-s001.zip › vaccines-1174508-supplementary.pdf]

Table S1. Antibody responses in a healthy self-vaccinated volunteer and healthy blood donors without anamnestic SARS-CoV-2 exposure.

An in-house ELISA was used to test antibody reactivity against the respective SARS-CoV-2 peptides and CMV-pp65 using plasma samples from one self-experimenting vaccinated volunteer (VV) obtained 36 days before vaccine administration (pre vac) and 19 days after vaccination with the peptides provided in **Table 1** (post vac) as well as serum samples from three healthy blood donors (HD). Final dilutions of all samples were 1:500 with peptide concentrations used at 35 µg/ml. Values considered as positive are indicated in bold. Abbreviations: env—envelope protein, HLA—human leukocyte antigen, nuc—nucleoprotein, OD—optical density, SARS-CoV-2—severe acute respiratory syndrome coronavirus 2.

	**SARS-CoV-2 env**		**SARS-CoV-2 nuc**		**SARS-CoV-2 nuc**		**CMV-pp65 363–373**		**CMV-pp65 510–524**	
HLA-	DR		DR		DR		A*01		DR	
Peptide ID	env-DR-1		nuc-DR-1		nuc-DR-2		CMV-A1-1		CMV-DR-1	
	OD x 10^3									
	IgG	IgM	IgG	IgM	IgG	IgM	IgG	IgM	IgG	IgM
VV (pre vac)	19	41	6	13	21	13	6	10	32	49
VV (post vac)	12	44	23	12	43	17	20	12	247	58
HD1	64	128	68	68	49	96	115	60	518	156
HD2	77	169	112	36	74	146	405	49	**1550**	146
HD3	30	441	16	67	25	69	383	63	**1457**	149

Table S2. Antibody responses in a healthy self-vaccinated volunteer and healthy blood donors with confirmed and without SARS-CoV-2 exposure.

An in-house ELISA was used to test antibody reactivity against SARS-CoV-2 proteins or peptides using plasma samples from one self-experimenting vaccinated volunteer (VV) obtained before (pre) or at given time points after vaccination with the peptides shown in **Table 1**. Of note, the sample VV 31 was drawn 31 days after the first vaccination with peptides shown in **Table 1** and 3 days after the second vaccination with the peptides shown in **Table 2**, respectively. Further, serum samples from healthy blood donors (HD) was tested, either from HD previously confirmed as SARS-CoV-2-positive (HD-CoV+) in medical routine diagnostics (qRT-PCR) or obtained already before the pandemic (HD1 to HD6). Recombinant proteins (SinoBiological, Beijing, China) and peptides were used (peptide sequences are specified in **Table 1 & 2**). Final dilutions of all samples were 1:500 with peptide concentrations used at 1 μg/ml. Values considered as positive are indicated in bold. Peptide sequences not mentioned elsewhere: spi-DR-3 (SARS-CoV-2, spike glycoprotein; AA: 446–460): GGNYNYLYRLFRKSN, spi-DR-5 (SARS-CoV-2, spike glycoprotein; AA: 458–472): KSNLKPFERDISTEI, spi-DR-6 (SARS-CoV-2, spike glycoprotein; AA: 338–352): FGEVFNATRFASVYA. Abbreviations: AA—amino acid; RBD—receptor-binding domain, rec.—recombinant, n.a.—not applicable, nuc.—nucleoprotein, OD—optical density, SARS-CoV-2—severe acute respiratory syndrome coronavirus 2.

	Days post Vaccination	Ig	rec. RBD Protein	rec. Spike-1 Protein	rec. nuc. Protein	spi-DR-1	spi-DR-2	spi-DR-3	spi-DR-5	spi-DR-6	nuc-DR-3	nuc-DR-9
OD x 10^3												
VV	pre	M	68	31	73	65	71	40	45	81	37	41
		G	96	53	59	62	76	43	39	46	45	35
VV	19	M	70	34	57	68	71	42	41	74	35	41
		G	94	47	52	62	70	43	41	34	40	31
VV	31	M	55	23	59	63	59	41	43	75	36	40
		G	80	49	56	83	63	37	31	28	31	29
HD-CoV+	n.a.	M	**1645**	**1614**	283	213	103	108	104	177	43	180
		G	**2410**	**2586**	**2588**	176	199	100	115	115	127	125

	Days post Vaccination	Ig	rec. RBD Protein	rec. Spike-1 Protein	rec. nuc. Protein	spi-DR-1	spi-DR-2	spi-DR-3	spi-DR-5	spi-DR-6	nuc-DR-3	nuc-DR-9
OD x 10^3												
HD1	n.a.	M	236	132	262	605	259	273	211	145	183	270
		G	347	121	**1944**	106	226	67	110	164	127	121
HD2	n.a.	M	142	94	167	452	471	316	211	226	228	96
		G	322	106	245	100	191	80	96	105	90	118
HD3	n.a.	M	289	205	385	277	324	148	255	171	179	189
		G	103	56	79	61	130	59	50	61	65	61
HD4	n.a.	M	112	80	165	265	333	237	134	108	153	89
		G	494	151	274	131	325	105	180	221	193	226
HD5	n.a.	M	366	250	498	759	385	509	425	228	297	211
		G	308	131	204	110	286	79	136	189	160	173
HD6	n.a.	M	359	293	495	394	895	288	329	272	279	365
		G	490	306	468	227	425	168	281	260	485	281
